# Twenty-three medication-taking traits and stroke: A comprehensive Mendelian randomization study

**DOI:** 10.3389/fcvm.2023.1120721

**Published:** 2023-03-20

**Authors:** Wenbo Shao, Taozhi Li, Yukun Wang, Shizhe Shan, Haiyu Zhang, Yanxing Xue

**Affiliations:** ^1^ Department of Cardiology, Guang'anmen Hospital, China Academy of Chinese Medical Sciences, Beijing, China; ^2^ Department of Oncology, Xiyuan Hospital, China Academy of Chinese Medical Sciences, Beijing, China; ^3^ Graduate School of Beijing University of Chinese Medicine, Beijing, China; ^4^ Department of Geratology, Guang'anmen Hospital, China Academy of Chinese Medical Sciences, Beijing, China

**Keywords:** medication categories, stroke, ischemic categories, hemorrhagic categories, Mendelian randomization, causal effect

## Abstract

**Background:**

Certain medication categories may increase the risk of stroke. Nonetheless, the evidence regarding the causal relationship of medication-taking in promoting stroke and subtypes is deficient.

**Methods:**

We evaluated the causal effect of a genetic predisposition for certain medication categories on stroke and subtypes (ischemic and hemorrhagic categories) by a two-sample Mendelian randomization (MR) analysis. Data for 23 medication categories were gathered from a genome-wide association study (GWAS) involving 318,177 patients. The Medical Research Council Integrative Epidemiology Unit Open GWAS database and the FinnGen consortium were used to gather GWAS data for stroke and subtypes. Inverse variance weighted, MR-Egger, and weighted median were used for the estimation of causal effects. Cochran's *Q* test, MR-Egger intercept test, and leave-one-out analysis were used for sensitivity analyses.

**Results:**

Ten medication categories were linked to a high stroke risk. Nine categories were linked to a high-risk ischemic stroke. Five categories were associated with small vessel ischemic stroke. Nine categories were positively associated with large artery atherosclerotic ischemic stroke. Three categories causally increased the possibility of cardioembolic ischemic stroke. Four categories were associated with intracerebral hemorrhage. Four categories were associated with nontraumatic intracranial hemorrhage. Three categories were causally associated with subarachnoid hemorrhage (SAH). Four categories were associated with the combination of SAH, unruptured cerebral aneurysm, and aneurysm operations SAH.

**Conclusions:**

This study confirms that some medication categories lead to a greater risk of strokes. Meanwhile, it has an implication for stroke screening as well as direct clinical signiﬁcance in the design of conduction of future randomized controlled trials.

## Introduction

As a common cerebrovascular accident, stroke has sparked numerous conversations. It not just hampers the normal life of patients and their families but imposes an additional economic load on the society as well (1). By the next decade, stroke-related cases and deaths are expected to roar by approximately 23 million and 7.8 million, respectively (2). As we all know, stroke is a multifactorial disease. It may be caused either by uncontrollable factors, like age and genes, or by such controllable factors as hypertension, diabetes mellitus, hyperlipidemia, smoking, obesity, and alcohol consumption (1). Approximately 7 out of 10 cases of stroke are ischemic stroke (IS), and the rest fall into the categories of hemorrhagic strokes and subarachnoid hemorrhage (SAH) (3).

Medication plays a vital part in preventing and treating diseases of all sorts. Nevertheless, drugs prove to be a mixed blessing. Each therapeutic-drug dose can trigger off unexpected medication reactions in patients, which are usually known but sometimes unknown (4). Opioids, for example, can alleviate pain, but the side effects of these drugs are almost worse than the pain that they block. Long-term use will take a toll on multiple systems such as the nervous, digestive, immune, and endocrine systems in the whole body (5–7). Furthermore, the public are at risk of multiple diseases due to the lack of physical exercise and unhealthy lifestyles (8), subsequently receiving multidrug therapy. Sometimes, the growing complexity of medical treatment is a headache for doctors, especially for residents and interns. Medicines, whether on prescription or brought over the counter, have the chance of increasing the risk of stroke onset (9). Antidepressants have been linked to a considerably higher risk of stroke incidence [hazard ratio (HR) 1.56; 95% confidence interval (CI): 1.50–1.61] (10). Therefore, knowledge of unexpected medication effects, maximizing the benefit of drugs and protecting the health of patients is of paramount importance. Considering stroke is the second worldwide cause of deaths (11), illustrating the probable association between common exposures and stroke is of paramount importance. Due to the bias and reverse causality inherent, the causal role between medication-taking and stroke has not been systematically examined.

The application of Mendelian randomization (MR) can allow us to explore the causal relationship of the risk factors concerning the disease *via* genetic variants indexing exposure, thus effectively evading such biases (12). Speciﬁcally, this approach overcomes the reverse causality bias due to unmodifiable genotype (12). It is assumed that exposure-associated genetic variants undergo random assortment at conception; genetic variants that adjust medication-taking could be utilized as an instrumental variable (IV) for the assessment of the impact of medication categories on the incidence of apoplexy. This study aimed to reveal the causal association between medication-taking and stroke *via* the two-sample MR approach.

## Materials and methods

### Study design

**Figure 1:**
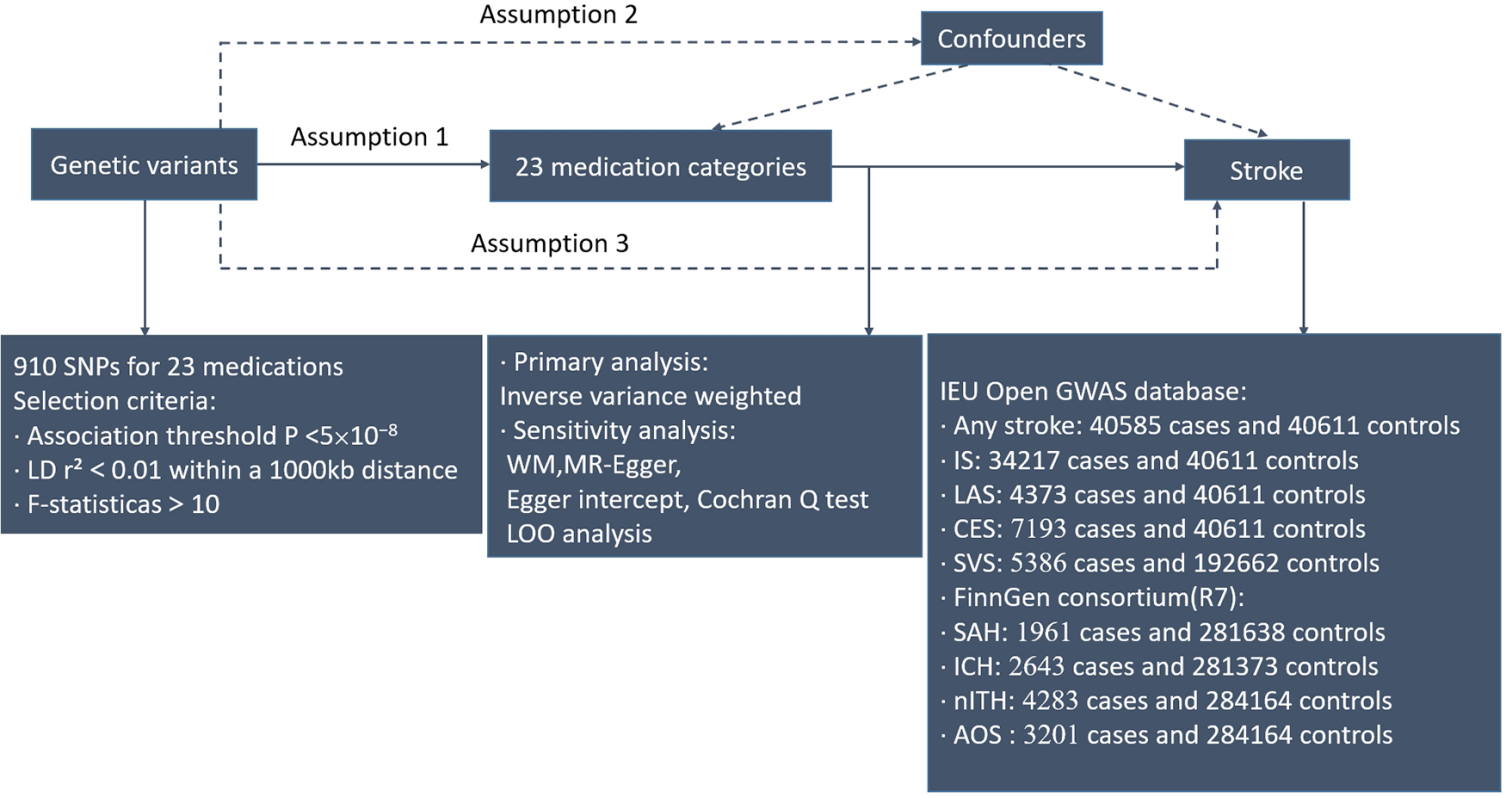
Workflow of Mendelian randomization study that shows causality between medication categories and strokes. Assumption 1, genetic variants are significantly linked to exposure; Assumption 2, genetic variants are not related to confounding factors; Assumption 3, genetic variants exert effects on the results *via* the exposure of interest. IEU, integrative epidemiology unit; IS, ischemic stroke; IVW, inverse variance weighted; LAS, large artery atherosclerotic ischemic stroke; CES, cardioembolic ischemic stroke; SVS, small vessel ischemic stroke; SAH, subarachnoid hemorrhage; ICH, intracerebral hemorrhage; nITH, nontraumatic intracranial hemorrhage; AOS, a combination of SAH, unruptured cerebral aneurysm, and aneurysm operations SAH; SNPs, single-nucleotide polymorphisms; LD, linkage disequilibrium; WM, weighted median; LOO, leave-one-out.

Our objective was to estimate the causal association between 23 medication-taking traits and the incidence of stroke adopting genome-wide association study (GWAS) summary statistics by a two-sample MR design. This instrumental variable analysis in the random assignment of single-nucleotide polymorphisms (SNPs) is parallel to randomized controlled trials (RCTs) (the impact of confounders like age and gender was not incorporated). In addition, this study was performed on the condition that three key assumptions were met: (i) Genetic instruments have strong predictive power for the exposure of interest (*p* < 5 × 10^−8^); (ii) Genetic instruments are not related to potential confounding factors (no statistical inference); and (iii) Interdependency exists between genetic instruments and the results, taking into account exposure and confounders. (Only through the risk factors do genetic instruments exert impact on the results.) (13) All statistical analyses in this study were run in R software (4.2.1) *via* the TwoSampleMR packages. The study overview was presented in Figure 1.

### Exposure to GWAS-23 medication-taking traits

After the exclusion of participants taking case drugs and similar drugs, a grand total of 23 case–control medication categories were included in this study. In any case, this should be explained. GWAS data for 23 medication-taking traits were derived from a case–control GWAS meta-analysis conducted among 318,177 United Kingdom Biobank (UKB) study participants from the UK Biobank (14). GWAS data of medication-taking traits were defined as the record of self-reported regular medication and health supplements taken weekly, monthly, or 3-monthly. Scholars coded these related drug and health supplement data in 6,745 categories. In total, 1,809 drug classes were taken by at least 10 participants among 6,745 medication categories. The active ingredients corresponding to these 1,809 categories were unearthed through online information, Drugs (https://www.drugs.com/), and NetDoctor (https://www.netdoctor.co.uk/). They were then categorized using the Anatomical Therapeutic Chemical (ATC) Classification System (15). More detailed information about the GWAS data can be obtained from the study of Wu et al. (14).

IVs associated with medication traits were identified by rigorous screening conditions from multiple perspectives. After a series of GWASs and post-GWAS analysis, SNPs were reported significantly associated as the candidate IVs across 23 medication traits ultimately [*p* < 5  ×  10^−8^, linkage disequilibrium (LD) r^2^ < 0.01]. The F-statistics of the selected IVs were all in excess of the conventional value of weak instruments of F-statistic/10, indicating that the IVs had a strong potential to predict 23 medication categories (16). Next, we extracted medication category-associated SNPs from the outcome and discarded SNPs associated with the outcome (*p* < 5  ×  10^−8^). We further harmonized SNPs for exposure and outcome, and palindromic effects and allelic inconsistent SNPs were removed (e.g., A/G vs. A/C). Details of the 910 SNPs are presented in Supplementary Table S1.

### GWAS summary data for stroke

Stroke, an acute cerebrovascular disease, is a rapidly progressing sign of focal (or global) disturbance of cerebral function, which mostly presents with sudden onset, rapid onset of limited, or diffuse brain dysfunction, with the clinical symptoms lasting more than 24 h (17). It is usually divided into two categories based on clinical and radiographic standard: ischemic and hemorrhagic. All summary-level data for stroke and ischemic stroke (all subtypes) in primary analysis were acquired from the publicly available Medical Research Council Integrative Epidemiology Unit (IEU) Open GWAS database (18). More detailed information about the sample characterization, statistical analysis, and genotyping can be found in the original publication (19). In brief, we gathered relevant stroke data from a large GWAS meta-analysis that included 29 genome-wide association studies with solely European individuals, containing a total of 40,585 cases of stroke and 34,217 cases of IS, with 40,611 controls. In accordance with the TOAST classification of stroke, IS participants were subdivided into three types in focus of infarction: large artery atherosclerotic (*n* = 4,373 cases/40,611 controls), cardioembolic (*n* = 7,193 cases/40,611 controls), and small vessel (*n* = 5386 cases/192,662 controls) in this meta-analysis, represented by LAS, CES, and SVS, respectively (19, 20). For hemorrhagic stroke, according to the FinnGen consortium (21), four hemorrhagic stroke outcomes were exhibited, including SAH (1,961 cases, and 281,638 controls), intracerebral hemorrhage (ICH, 2,643 cases and 281,373 controls), nontraumatic intracranial hemorrhage (nITH, 4,283 cases and 284,164 controls), and a combination of SAH, unruptured cerebral aneurysm, and aneurysm operations SAH (AOS) (3,201 cases and 284,164 controls) (21). Aneurysm operations in AOS are endovascular or surgical procedures to intracerebral aneurysms. AOS was involved in hemorrhagic stroke outcomes due to the end-point attribute of SAH in AOS. According to how FinnGen defines the end point, nITH can be viewed as equivalent to hemorrhagic stroke based on the exclusion of other brain hemorrhages (21). As a result, we extracted summary statistics from the R7 release of the FinnGen consortium. In addition, the participants in our MR analyses were all of European descent. GWASs employed in the study are specified in Table 1.

**Table 1:** Details of the GWASs included in the Mendelian randomization.

Consortium	Phenotype	Cases	Source
A meta-analysis of GWAS	23 medication categories	318,177	https://www.nature.com/articles/s41467-019-09572-5
IEU	Stroke	40,585	https://gwas.mrcieu.ac.uk/
IEU	IS	34,217	https://gwas.mrcieu.ac.uk/
IEU	CES	7,193	https://gwas.mrcieu.ac.uk/
IEU	LAS	4,373	https://gwas.mrcieu.ac.uk/
IEU	SVS	5,386	https://gwas.mrcieu.ac.uk/
FinnGen consortium	ICH	2,643	https://www.finngen.fi/en
FinnGen consortium	SAH	1,961	https://www.finngen.fi/en
FinnGen consortium	nITH	4,183	https://www.finngen.fi/en
FinnGen consortium	AOS	3,201	https://www.finngen.fi/en
IEU	Hypertension	11,863	https://gwas.mrcieu.ac.uk/
DIAGRAM	Type 2 diabetes	12,171	https://gwas.mrcieu.ac.uk/
GLGC	Total cholesterol	Continuous variable	https://gwas.mrcieu.ac.uk/
IEU	GORD	129,080	https://gwas.mrcieu.ac.uk/

IEU, integrative epidemiology unit; IS, ischemic stroke; LAS, large artery atherosclerotic ischemic stroke; CES, cardioembolic ischemic stroke; SVS, small vessel ischemic stroke; SAH, subarachnoid hemorrhage; ICH, intracerebral hemorrhage; nITH, nontraumatic intracranial hemorrhage; AOS, a combination of SAH, unruptured cerebral aneurysm, and aneurysm operations SAH; GORD, gastroesophageal reflux disease; GLGC, Global Lipids Genetics Consortium.

### Statistical analysis

We employed some types of MR approaches [inverse variance weighted (IVW), MR-Egger, and WM] to determine MR estimates of medication-taking for stroke after harmonizing the effect alleles across the GWASs of 23 medication categories and stroke was completed. As a method for meta-summarization of effects at multiple loci, IVW was used as a primary analytical method in a relatively ideal state and its application assumes that all SNPs are valid and completely independent of each other (22, 23). Moreover, several other MR approaches including MR-Egger and WM have been utilized for the detection of the robustness of study results in a wider range of scenarios. As the median of the distribution function obtained by sorting all individual SNP effect values by weight, WM permits fewer than 50% of SNPs to be invalid, yet MR-Egger permits pleiotropy of all SNPs, but it assumes that the effect of genetic variation pleiotropy on the outcome is independent of the effect of genetic variation on exposure factors. In other words, MR-Egger was able to offer a test for heterogeneity and pleiotropy for all SNPs in the existence of horizontal heterogeneity (24, 25). *p* < 0.002 (Bonferroni correction *p *= 0.05/23 sets of exposures) is considered to show a remarkable causal relationship. Taking inconsistency into account, the study adopted a tightened instrument *p*-value threshold (26).

In addition, specific sensitivity analyses were used to evaluate and adjust any potential heterogeneity and pleiotropy to satisfy relevant key assumptions of our MR study design. Cochran's *Q* statistic was employed to detect heterogeneity. With an aim to guarantee no directional pleiotropy for the linkage between medical traits and stroke of all subcategories, the *p-*value of the Cochran *Q* test should exceed 0.05 (27). Furthermore, we conducted the MR-Egger intercept tests to appraise the influence level of directional pleiotropy on risk estimates (25). The intercept value obtained from MR-Egger regression deviating considerably from null (*p *< 0.05) was employed for indicating the presence of horizontal pleiotropy (28, 29). For further interpretation, we performed the leave-one-out (LOO) analysis, which assesses whether the results are strongly influenced by each exposure-associated SNP and then repeat the IVW analysis in turn by discarding a SNP (28).

### GWAS summary data for risk factors

To demonstrate the mediating effect of certain medication categories on stroke *via* some risk factors, we calculated some specific diseases of drug indications representative as potential mediators for further MR analysis, namely, hypertension, type 2 diabetes, gastroesophageal reflux disease (GORD), and total cholesterol. GWASs employed in the study are also specified in Table 1.

## Results

### Mendelian randomization and sensitivity analysis between 23 medication categories and stroke

**Figure 2:**
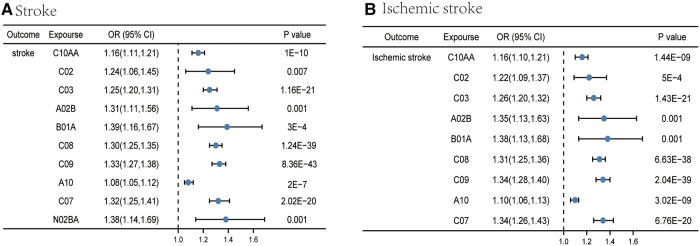
Causal effects from certain medication categories on stroke (**A**) and ischemic stroke (**B**). The IVW approach was utilized for the summary of the MR estimates. C10AA, HMG CoA reductase inhibitors; C02, antihypertensives; C03, diuretics; A02B, drugs for peptic ulcer and GORD; B01A, antithrombotic agents; C08, calcium channel blockers; C09, agents acting on the renin-angiotensin system; A10, drugs used in diabetes; C07, beta blocking agents; N02BA, salicylic acid and its derivatives.

Among the medication categories tested, by using medication-related SNPs, IVW initially screened out 10 medication categories (HMG CoA reductase inhibitors, antihypertensives, diuretics, drugs for peptic ulcer and GORD, antithrombotic agents, calcium channel blockers, agents acting on the renin-angiotensin system, drugs used in diabetes, beta blocking agents, and salicylic acid and its derivatives) significantly associated with stroke (Supplementary Tables S2, S3 and Figure 2A), implying a high-risk function on stroke. Meanwhile, a consistent direction in MR-Egger and weighted median supported the robustness of the result.

In addition, the MR-Egger intercept testing suggested no sign of a possible horizontal pleiotropy effect (Supplementary Table S4). However, with the exception of a small number of medication categories, defined as antihypertensives and drugs for peptic ulcer and GORD, heterogeneity was examined in Cochran's *Q* test analysis between categories mentioned above and stroke, including HMG CoA reductase inhibitors (*p* for Cochran's *Q* = 1.94 × 10^−6^), diuretics (*p* for Cochran's *Q* = 1.78 × 10^−12^), antithrombotic agents (*p* for Cochran's *Q* = 9.70 × 10^−5^), calcium channel blockers (*p* for Cochran's *Q* = 1.25 × 10^−5^), agents acting on the renin-angiotensin system (*p* for Cochran's *Q* = 2.69 × 10^−14^), drugs used in diabetes (*p* for Cochran's *Q* = 0.01), beta blocking agents (*p* for Cochran's *Q* = 2.36 × 10^−6^), and salicylic acid and its derivatives (*p* for Cochran's *Q* = 7.55 × 10^−5^) (Supplementary Table S5). Despite the existence of heterogeneity in some outcomes, MR estimates were not invalidated as random-effect IVW in this research, which could act as a counterbalance to pooled heterogeneity.

### Mendelian randomization and sensitivity analysis between 23 medication categories and ischemic stroke

For IS, IVW yielded the estimates of the effect of nine medication categories (HMG CoA reductase inhibitors, antihypertensives, diuretics, drugs for peptic ulcer and GORD, antithrombotic agents, calcium channel blockers, agents acting on the renin-angiotensin system, drugs used in diabetes, and beta blocking agents) on IS (Supplementary Tables S6, S7 and Figure 2B), as shown in the figure. Similar risk estimates were gained using the MR-Egger regression and WM approaches, though sometimes the association was of no statistical significance. *p* values of the MR-Egger intercept testing all exceeded 0.05, suggesting the absence of horizontal pleiotropy (Supplementary Table S8). At the same time, we detected the heterogeneity among some medication categories, including HMG CoA reductase inhibitors (*p* for Cochran's *Q* = 2.91 × 10^−5^), diuretics (*p* for Cochran's *Q* = 6.08 × 10^−10^), calcium channel blockers (*p* for Cochran's *Q* = 4.89 × 10^−5^), agents acting on the renin-angiotensin system (*p* for Cochran's *Q* = 4.05 × 10^−15^), and beta blocking agents (*p* for Cochran's *Q* = 1.39 × 10^−6^) (Supplementary Table S9). Since the random-effects IVW were employed as the primary outcome, heterogeneity was at an acceptable level (30).

**Figure 3:**
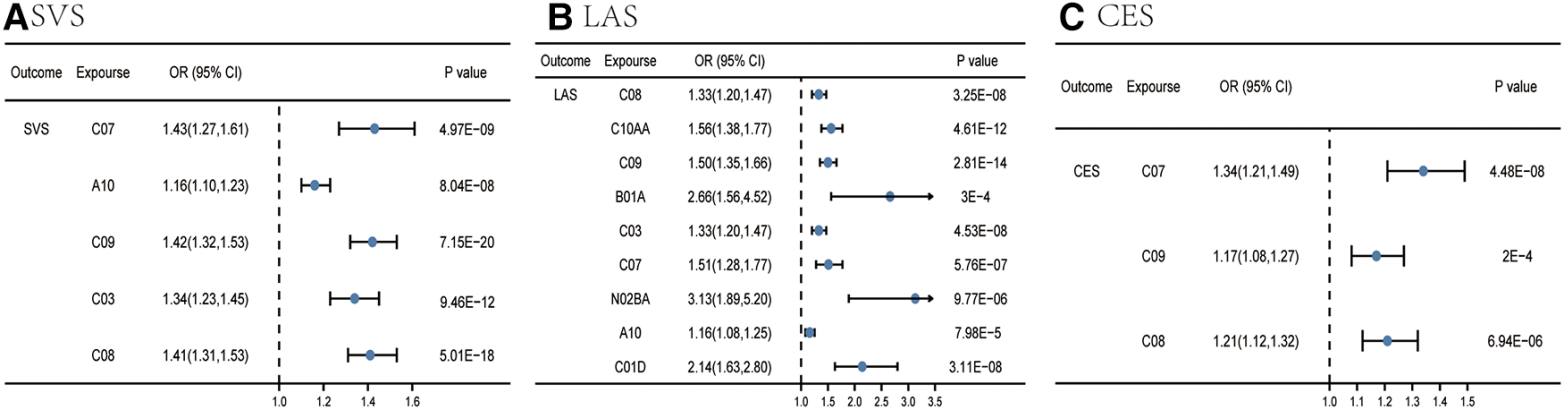
Causal effects from certain medication categories on SVS (**A**), LAS (**B**), and CES (**C**). The IVW approach was utilized for the summary of the MR estimates. LAS, large artery atherosclerotic ischemic stroke; CES, cardioembolic ischemic stroke; SVS, small vessel ischemic stroke; C10AA, HMG CoA reductase inhibitors; C03, diuretics; B01A, antithrombotic agents; C08, calcium channel blockers; C09, agents acting on the renin-angiotensin system; A10, drugs used in diabetes; C07, beta blocking agents; N02BA, salicylic acid and derivatives; C01D, vasodilators used in cardiac diseases.

As for subtypes of IS, IVW preliminarily identified six categories significantly associated with SVS, which were beta blocking agents, drugs used in diabetes, agents acting on the renin-angiotensin system, diuretics, HMG CoA reductase inhibitors, and calcium channel blockers. Nevertheless, pleiotropy was observed in the MR-Egger intercept analysis between HMG CoA reductase inhibitors and SVS (*p* = 0.002) (Supplementary Table S12). Meanwhile, the result of MR-Egger suggested that no causal relation existed between HMG CoA reductase inhibitors and SVS [odds ratio (OR) = 1.00, 95% CI = 0.839–1.192, *p* = 0.999]. Therefore, we would regard this as an insignificant estimate. Eventually, only five of them met the criteria involved in the development of SVS (Supplementary Tables S10, S11 and Figure 3A), including beta blocking agents, drugs used in diabetes, agents acting on the renin-angiotensin system, diuretics, and calcium channel blockers. In other words, these five categories were linked to a high SVS risk. The *Q* test outcome demonstrated heterogeneity between beta blocking agents and SVS (*p* for Cochran's *Q* = 0.0001), agents acting on the renin-angiotensin system and SVS (*p* for Cochran's *Q* = 4.12 × 10^−5^), diuretics and SVS (*p* for Cochran's *Q* = 8.25 × 10^−5^), and calcium channel blockers and SVS (*p* for Cochran's *Q* = 0.0004) (Supplementary Table S13).

For LAS, nine categories (calcium channel blockers, HMG CoA reductase inhibitors, agents acting on the renin-angiotensin system, antithrombotic agents, diuretics, beta blocking agents, salicylic acid and its derivatives, drugs used in diabetes, and vasodilators used in cardiac diseases) were associated with the advance of LAS in the IVW analysis (Supplementary Tables S14, S15 and Figure 3B). Other two MR methods yielded a consistent direction, supporting the robustness of the result. Pleiotropy was observed in the MR-Egger intercept analysis between beta blocking agents and LAS (*p* = 0.02); the result of MR-Egger suggested that beta blocking agents had evidence of causality on LAS (OR = 2.98, 95% CI = 1.67–5.30, *p* = 0.0005) (Supplementary Table S16). The *Q* test result indicated heterogeneity between calcium channel blockers and LAS (*p* for Cochran's *Q* = 0.03), HMG CoA reductase inhibitors and LAS (*p* for Cochran's *Q* = 0.0009), agents acting on the renin-angiotensin system and SVS (*p* for Cochran's *Q* = 1.10 × 10^−8^), antithrombotic agents and LAS (*p* for Cochran's *Q* = 0.002), diuretics and LAS (*p* for Cochran's *Q* = 0.02), beta blocking agents and LAS (*p* for Cochran's *Q* = 0.0001), and salicylic acid and its derivatives and LAS (*p* for Cochran's *Q* = 0.02) (Supplementary Table S17).

For CES, IVW identified three categories significantly associated with a high risk of CES (Supplementary Tables S18, S19 and Figure 3C), including beta blocking agents, agents acting on the renin-angiotensin system, and calcium channel blockers. Pleiotropy was also observed in the MR-Egger intercept analysis between agents acting on the renin-angiotensin system and CES (*p* = 0.006); the result of MR-Egger estimated that agents acting on the renin-angiotensin system had the causality on CES (OR 1.65, 95% CI 1.27–2.13, *p* = 0.0002) (Supplementary Table S20). The *Q* test outcome showed heterogeneity between agents acting on the renin-angiotensin system and CES (*p* for Cochran's *Q* = 8.28 × 10^−7^), calcium channel blockers, and CES (*p* for Cochran's *Q* = 0.0008) (Supplementary Table S21).

### Mendelian randomization and sensitivity analysis between 23 medication categories and hemorrhagic stroke

**Figure 4:**
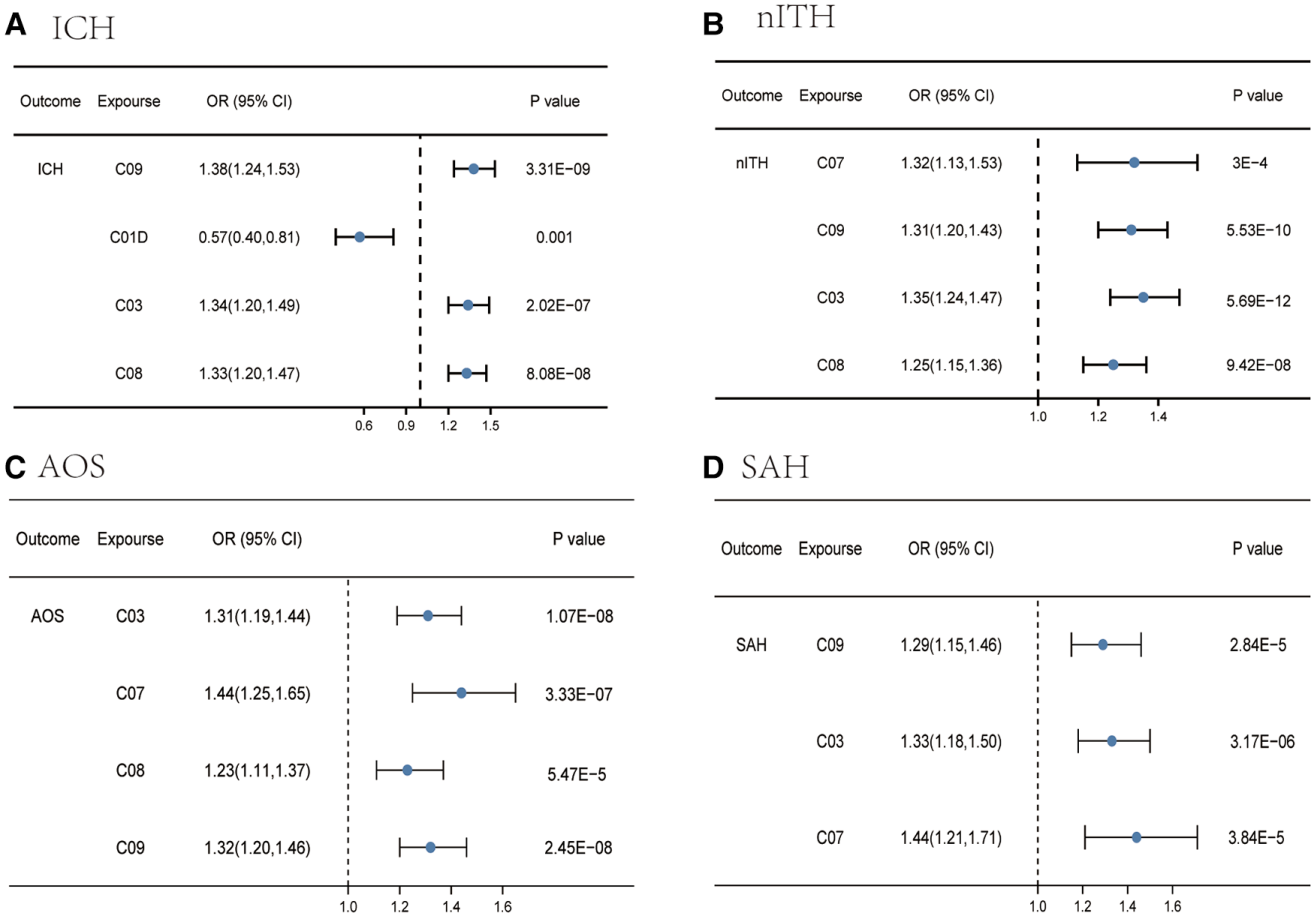
Causal effects from certain medication categories on ICH (**A**), nITH (**B**), AOS (**C**), and SAH (**D**). The IVW approach was utilized for the summary of the MR estimates. SAH, subarachnoid hemorrhage; ICH, intracerebral hemorrhage; nITH, nontraumatic intracranial hemorrhage; AOS, a combination of SAH, unruptured cerebral aneurysm, and aneurysm operations SAH; C03, diuretics; C08, calcium channel blockers; C09, agents acting on the renin-angiotensin system; C07, beta blocking agents; C01D, vasodilators used in cardiac diseases.

For ICH, IVW identified four categories (agents acting on the renin-angiotensin system, vasodilators used in cardiac diseases, diuretics, and calcium channel blockers) significantly associated with ICH (Supplementary Tables S22, S23 and Figure 4A), as the figure shows. The MR-Egger intercept testing showed no signs of horizontal pleiotropy effect (Supplementary Table S24). Heterogeneity was also detected between agents acting on the renin-angiotensin system and ICH (*p* for Cochran's *Q* = 0.03), and diuretics and ICH (*p* for Cochran's *Q* = 0.04) (Supplementary Table S25).

For nITH, four categories were linked to a high nITH risk in the IVW analysis (Supplementary Tables S26, S27 and Figure 4B), including beta blocking agents, agents acting on the renin-angiotensin system, diuretics, and calcium channel blockers. Notably, we did not detect evidence of a causal association in two categories [beta blocking agents (*p* = 0.02) and agents acting on the renin-angiotensin system (*p* = 0.17)] *via* the MR-Egger analysis, but a consistent direction made the outcome convincing. Meanwhile, the WM yielded a consistent direction and magnitude, with all *p* values of four categories greater than 0.05, supporting the robustness of the result. Although we observed the presence of horizontal pleiotropy between calcium channel blockers and nITH in IVW estimates, the significant causal association was verified by MR-Egger regression (Supplementary Table S28). In addition, we can observe heterogeneity in the *Q* test analysis between beta blocking agents and nITH (*p* for Cochran's *Q* = 9.92 × 10^−6^), and agents acting on the renin-angiotensin system and nITH (*p* for Cochran's *Q* = 0.009) (Supplementary Table S29). Nevertheless, MR estimates were not invalidated as random-effect IVW in this research by the presence of heterogeneity.

For AOS, IVW identified four categories (diuretics, beta blocking agents, calcium channel blockers, and agents acting on the renin-angiotensin system) significantly associated with this combination (Supplementary Tables S30, S31 and Figure 4C). The direction was consistent in MR-Egger and weighted median, supporting the robustness of the result. The *p-*value for the MR-Egger intercept is >0.05 except for calcium channel blockers, indicating that no horizontal pleiotropy existed (Supplementary Table S32). The positive causal estimates between genetic predisposition toward calcium channel blockers and AOS were still verified using MR-Egger analysis. In addition, despite the detection of heterogeneity in calcium channel blockers (*p* for Cochran's *Q* = 0.007) and agents acting on the renin-angiotensin system (*p* for Cochran's *Q* = 0.016) with AOS (Supplementary Table S33), the causal relations were still valid due to acceptable heterogeneity (30).

For SAH, IVW identified three categories significantly associated with SAH (Supplementary Tables S34, S35 and Figure 4D), including agents acting on the renin-angiotensin system, diuretics, and beta blocking agents, as can be seen from the figure. Other two MR methods yielded a consistent direction, supporting the robustness of the result. Beyond this, no trace of remarkable intercept indicated that there was no possible pleiotropy observed (Supplementary Table S36). No heterogeneity was detected in Cochran's *Q* test analysis (Supplementary Table S37).

### Risk factor analysis

To explore if the risk factors disturb the causal relationship between medical categories and stroke, we used MR approaches to evaluate the causality between stroke and relevant risk factors such as hypertension, type 2 diabetes, gastroesophageal reflux disease, and total cholesterol. As shown in Table 2, we found that total cholesterol level was significantly associated with stroke (*p *= 0.004), IS (*p *= 0.003), and LAS (*p *= 0.003). Meanwhile, the existence of causal effect GORD on stroke (4.58 × 10^−7^) and IS (1.72 × 10^−5^) was verified in IVW analysis. For hypertension and type 2 diabetes, no causal effects were observed from these two diseases on stroke and all subtypes, implying that the causality of certain medication categories on stroke phenotypes was not biased by potential risk factors.

**Table 2:** Risk factor analysis.

Exposure	Outcomes	Causal effect (95% CI)	*p-*value
GORD	IS	1.17 (1.09–1.27)	1.72 × 10^−5^
GORD	Stroke	1.19 (1.12–1.28)	4.58 × 10^−7^
Total cholesterol	Stroke	1.09 (1.03–1.16)	0.004
Total cholesterol	IS	1.11 (1.04–1.20)	0.003
Total cholesterol	LAS	1.28 (1.08–1.51)	0.003
Hypertension	Stroke	1.11 (0.94–1.32)	0.42
Hypertension	IS	1.10 (0.91–1.31)	0.32
Hypertension	SVS	1.10 (0.77–1.57)	0.59
Hypertension	LAS	1.57 (0.91–2.69)	0.09
Hypertension	CES	1.23 (0.81–1.87)	0.32
Hypertension	ICH	1.16 (0.63–2.13)	0.64
Hypertension	nITH	0.92 (0.57–1.50)	0.76
Hypertension	AOS	0.79 (0.45–1.39)	0.42
Hypertension	SAH	0.96 (0.48–1.95)	0.91
Type 2 diabetes	Stroke	1.04 (1.00–1.08)	0.06
Type 2 diabetes	IS	1.04 (1.00–1.09)	0.06
Type 2 diabetes	SVS	1.10 (0.97–1.24)	0.12
Type 2 diabetes	LAS	1.08 (0.92–1.27)	0.33

IS, ischemic stroke; LAS, large artery atherosclerotic ischemic stroke; CES, cardioembolic ischemic stroke; SVS, small vessel ischemic stroke; SAH, subarachnoid hemorrhage; ICH, intracerebral hemorrhage; nITH, nontraumatic intracranial hemorrhage; AOS, a combination of SAH, unruptured cerebral aneurysm, and aneurysm operations SAH; GORD, gastroesophageal reflux disease.

## Discussion

By means of a two-sample MR approach, GWAS data were analyzed with a view to comprehensively investigate the causal relations of some specific medication categories with the risk of stroke, ischemic categories, and hemorrhagic categories. So far as we know, this is the first study that applied a large-scale MR approach to a comprehensive evaluation of the possible causal relations between 23 medication-taking traits and stroke.

Many risk factors have been proposed to increase the incidence of stroke, like obesity, age, diabetes, dyslipidemia, smoking, and a sedentary lifestyle (31, 32). Previous studies had reported that some medication categories are proven to exert an influence on the incidence of stroke, including erythropoietin, bevacizumab, tamoxifen, and antipsychotics, among others (9). While different studies pointed to the potential influence of medication categories, the associated proof remains scarce to confirm the causal relations between the two. As is known, elderly persons regularly receive complex multidrug therapy due to multiple diseases, either acute or chronic. As a consequence, improper drug use poses a serious challenge to the health of patients, particularly for senior citizens (33, 34). Nevertheless, persuasive data are still lacking to specify the risks of a number of officially approved medicines on prescription. Less is known about whether certain medication categories might influence stroke due to insufficient data. Inspired by the medication-use GWAS analysis performed by Wu et al. (14), we devised a full-exposure MR study for causality assessment between medication categories and stroke with a view to maximize the benefit of drugs and stroke prevention. In contrast to large-scale prospective clinical trials, which are relatively impractical and require long-term observation, an MR study elucidates cause-and-effect relationships between drug categories and stroke in a timely and cost-effective manner.

Stroke, as an unwanted reaction to some specific medication categories, has triggered off a heavy burden on healthcare systems (35). Thus, knowledge of the effects taking into account such multiple factors as prescribed drugs, specific diseases, and metabolic pathways is of particular importance. The present study found clear proof that genetic liability for some specific medication-taking was causally linked to a greater risk of stroke and various subcategories, while genetic predisposition toward medication-taking of vasodilators used in cardiac diseases played a protective role in ICH advance. To elaborate, consistent direction and magnitude of influence estimates verified the causal relations *via* different MR approaches, IVW, WM, along with MR-Egger. We demonstrated that HMG CoA reductase inhibitors, antihypertensives, diuretics, drugs for peptic ulcer and GORD, antithrombotic agents, calcium channel blockers, agents acting on the renin-angiotensin system, drugs used in diabetes, beta blocking agents, and salicylic acid and its derivatives causally increased the risk for stroke. In the abovementioned list, all except salicylic acid and its derivatives causally increased the possibility of IS. In addition, this study demonstrated the causal relations between beta blocking agents, drugs used in diabetes, agents acting on the renin-angiotensin system, diuretics, calcium channel blockers, and increased SVS risk. Calcium channel blockers, HMG CoA reductase inhibitors, agents acting on the renin-angiotensin system, antithrombotic agents, diuretics, beta blocking agents, salicylic acid and its derivatives, drugs used in diabetes, and vasodilators used in cardiac diseases were linked to an increased LAS risk. Beta blocking agents, agents acting on the renin-angiotensin system, and calcium channel blockers were identified to play a positive role in CES development, implying a harmful function on CES. In addition, compared to vasodilators used in cardiac diseases playing a protective role in ICH, agents acting on the renin-angiotensin system, diuretics, and calcium channel blockers were associated with a high risk of ICH, and agents acting on the renin-angiotensin system, diuretics, and beta blocking agents eventually contribute to the incidence of SAH. Beta blocking agents, agents acting on the renin-angiotensin system, diuretics, and calcium channel blockers also served as catalysts for the incidence of nITH and AOS.

Of note, drugs for peptic ulcer and GORD causally increased the possibility of stroke and IS. It deserved to be discussed. Peptic ulcer and GORD are very common disorders, and proton pump inhibitors (PPIs), as one of the representative drugs, take action by performing consistent inhibitory effects on acid secretion (36). Widely used globally, the amount of PPIs used in the United States multiplied by 3.9% in 2021 compared with the equivalent period in1999 (37). However, the last few decades have seen the topic of a variety of adverse events associating chronic PPIs use rush into the forefront of the public, including the cardiovascular (CV) system, type 2 diabetes, cancer, and so on (38, 39). Meanwhile, whether PPI users suffer a higher risk of stroke draws concern among scholars. A variety of studies have reported inconsistent outcomes regarding the relations between PPIs and stroke. In a large study based on a 5.8-year follow-­up, Sehested et al. (40) revealed that PPI users have increased chances of developing first-time ischemic stroke and myocardial infarction (MI), especially those who use PPIs on a regular basis and on a high dosage, while in this study, confounders like lifestyle and indications of PPI therapies were not adjusted in the final analysis. However, Nguyen et al. (41) reported that the use of PPIs seemed not to be significantly associated with stroke after adjustment for lifestyle factors and indication, whereas the evidence came from an observational study, and the presence of additional residual and confounding factors were inevitable. This MR study revealed the causality between drugs for peptic ulcer, GORD, stroke, and IS by GWAS summary statistics through genetic instruments, which are free of the interference of confounders and reverse causation concerns.

A possible mechanism for this relationship is that in murine models, PPIs may serve as catalysts for a growing level of asymmetrical dimethylarginine (ADMA) (42) and reduce nitric oxide (NO) (43). There has been an increasing awareness that vascular cell proliferation and endothelial–leukocyte interactions are affected by the bioavailability of NO (44). The reduction in the bioavailability of NO invites endothelial dysfunction, subsequently, resulting in atherosclerosis over time. Second, the study has reported that PPIs may exert a reduction in vitamin B12 (45), which could promote homocysteine and hence elevate ADMA levels, triggering off endothelial dysfunction. Furthermore, the reasons for metabolic syndrome are manifold and varied, and the one that acts is PPI use (46), which subsequently raises the chance of stroke. The internal mechanism between PPI use and stroke remains to be proved through a series of studies.

For some other medication categories that are significantly associated with the development of stroke and its subtypes, we tend to live under the illusion that the drug itself has a direct causality on stroke and subtypes; obviously, it seems to fail to take into the fact that the drug-related diseases can also increase risk of stroke. For example, HMG CoA reductase inhibitors are the current mainstay of therapy for hyperlipidemia (47), which is one of the significant risk factors for stroke events. There is a clear association between hyperlipidemia and IS (48).

As a result, these findings highlighted that drug-related diseases, rather than medication, might be the root cause of increased risk of stroke. Notably, combining the MR result in risk factors analysis, GORD was significantly associated with stroke and IS, implying that GORD can also causally increase risk of stroke (OR 1.19, 95% CI 1.12–1.28, *p* = 4.58 × 10^−7^) and IS (OR 1.17, 95% CI 1.09–1.27, *p* = 1.72 × 10^−5^), and our accompanying MR analysis showed that drugs for peptic ulcer and GORD causally increased the possibility of stroke (OR 1.31, 95% CI 1.11–1.56, *p* = 0.002) and IS (OR 1.35, 95% CI 1.13–1.63, *p* = 0.001). In other words, GORD itself can exert a grave causal influence on stroke phenotypes, and so does the drug itself. Contrary to the influence caused by GORD on stroke, hypertension and type 2 diabetes did not affect the outcomes significantly according to our MR result, while a previous study (1) showed that these two may act as enzymes for higher stroke risk. Therefore, the extent to which diseases contribute to stroke outcomes varies, and further studies are required to identify the mechanisms of their mediation. Collectively, the purpose of this present MR study was to help doctors better comprehend the underlying stroke mechanism in patients with complications or otherwise and encourage doctors to inform patients about stroke prevention when taking some specific medications.

The advantages of the current study include the two-sample MR study design, the large-­scale GWAS sample size, multiple outcomes of stroke phenotypes, and multiple analyses involving genetic instruments, which had a significant association with 23 medication categories traits, to eliminate reverse causation, and residual confounding. Multiple methods and detailed analyses made our results more authentic.

However, due to various uncontrollable factors, the study inevitably has some limitations. First, our data source is based on GWAS summary statistics in individuals from Europe, which might invite the causal generalizability of our report to be further certified in other parts of the world. Second, we picked 23 medical traits because only these GWAS are available. As for the impact of other medication categories on stroke, additional work needs to be done to explore the mechanisms. Third, because of the application of a two-sample MR study based on a variety of sources, perhaps there exists unfathomable heterogeneity between studies (30). Finally, even if the MR method puts up an outstanding performance at the inference of causal association, we still admonish that the results of the MR study should be further verified in strongly proved randomized controlled trials to certify the presence of causality.

## Conclusions

Specifically speaking, we found that some specific medication categories were linked to a high risk of ischemic stroke, and other categories were connected to a substantially higher risk of hemorrhagic stroke. For example, 10 medication categories (HMG CoA reductase inhibitors, antihypertensives, diuretics, drugs for peptic ulcer and GORD, antithrombotic agents, calcium channel blockers, agents acting on the renin-angiotensin system, drugs used in diabetes, beta blocking agents, and salicylic acid and its derivatives) significantly associated with stroke. Notably, drugs for peptic ulcer and GORD increased the possibility of stroke and IS. This interesting finding should be further explored.

In summary, our findings can be of some reference to clinicians in the guidance of the conduction of future RCTs and have an implication for stroke screening and providing actionable strategies for the prevention of stroke.

## Data Availability

Publicly available datasets were analyzed in this study. These data can be found here: https://www.nature.com/articles/s41467-019-09572-5, https://gwas.mrcieu.ac.uk/, and https://www.finngen.fi/en.
